# EBV-associated gastric carcinoma in high- and low-incidence areas for nasopharyngeal carcinoma

**DOI:** 10.1038/sj.bjc.6605168

**Published:** 2009-07-14

**Authors:** T Boysen, M Mohammadi, M Melbye, S Hamilton-Dutoit, B Vainer, A V Hansen, J Wohlfahrt, J Friborg

**Affiliations:** 1 Department of Epidemiology Research, Statens Serum Institut, Artillerivej 5, 2300 Copenhagen S, Denmark; 2 Department of Pathology, Rigshospitalet, Copenhagen University Hospital, Blegdamsvej 9, 2100 Copenhagen O, Denmark; 3 Institute of Pathology, Aarhus University Hospital, Norrebrogade 44, 8000 Aarhus C, Denmark

**Keywords:** Epstein–Barr virus, gastric carcinoma, nasopharyngeal carcinoma

## Abstract

**Background:**

Approximately 10% of gastric carcinomas are associated with Epstein–Barr virus (EBV). The Inuit in Greenland have a high incidence of EBV-associated nasopharyngeal carcinoma.

**Methods:**

We conducted a population-based case–control study comparing gastric carcinomas in Greenland and in Denmark.

**Results:**

The prevalence rate of EBV-associated gastric carcinomas was 8.5% in both populations.

**Conclusion:**

The findings of this study argue against a general susceptibility to EBV-associated carcinomas among the Inuit.

Approximately 8–10% of gastric carcinomas worldwide are associated with Epstein–Barr virus (EBV) infection, making gastric carcinomas the most frequent EBV-associated malignancy (Burgess *et al*, 2002; Hjalgrim *et al*, 2008). The involvement of EBV in gastric carcinomas is based on the specific presence of viral gene products such as EBV-encoded small RNA (EBER) in tumour cells, but not in the surrounding non-neoplastic epithelium, and the presence of clonal EBV in tumour cells. (Shibata and Weiss, 1992; Imai *et al*, 1994). However, the precise aetiological role of EBV in this cancer and the mechanisms leading to EBV infection of the gastric epithelium are still unknown.

Undifferentiated nasopharyngeal carcinoma (NPC) is rare in most parts of the world, but is frequent in Southern China, North Africa and among the Inuit in the Arctic. The association between EBV and undifferentiated NPC has been known for the last 40 years, and is now firmly established (IARC working group, 1997; Hjalgrim *et al*, 2008). A study among the Chinese indicated that EBV-associated gastric carcinomas are more common in high- as compared with low-incidence regions of NPC, suggesting that these share risk factors (Hao *et al*, 2002). Not only are the Greenlandic Inuit an NPC high-risk population but they also have the world's highest incidence of salivary gland carcinomas, the majority being of EBV-associated lymphoepithelial carcinoma type (Hamilton-Dutoit *et al*, 1991; Friborg *et al*, 2003). Remarkably, the incidence of gastric carcinomas among the Inuit is increasing in contrast to the pattern seen elsewhere; the reasons for this increase are unknown (Friborg *et al*, 2003; Parkin *et al*, 2005; Alberts *et al*, 2006).

To determine whether the proportion of EBV-associated gastric carcinomas differs in the Inuit compared with that in the Danish population, we conducted a population-based comparative study of patients with gastric carcinoma in Greenland and Denmark.

## Materials and methods

The proportion of EBV-associated gastric carcinomas in Greenland and Denmark was examined in a case–control design using the Danish Cancer Registry (DCR), the Danish Civil Registration System and the Danish Pathology Database. All individuals in Denmark and Greenland are registered in the Civil Registration System (CRS). The CRS was established in Denmark on 1 April 1968 and in Greenland on 1 June 1972, when all persons alive and resident were registered and assigned a unique personal identification number (the person number). Information on cancer was retrieved from the DCR. Reporting of cancer cases to the DCR is mandatory in both Greenland and Denmark, and the coverage of the Cancer Registry in Denmark has shown to be 95–98% (Storm *et al*, 1997). Since 1975, the main source of information in the registry in Greenland has been notifications from physicians diagnosing and treating cancer patients, supplemented by the information obtained from pathology reports and death certificates. Information in the registry related to cancer cases in Greenland before 1975 was obtained from a study based on review of patient records from all hospitals in Greenland (Nielsen, 1986; Storm *et al*, 1997). The proportion of cases based on histological verification during the period 1973–1997 was 93 and 85% in Denmark and Greenland, respectively (Friborg *et al*, 2003).

All gastric carcinomas diagnosed during the period 1973–2002 in individuals born in Greenland were identified in the Danish Cancer Register. The 145 cases thus found were screened using the Danish Pathology Database, and tumour material was identified in 117 and frequency matched with gastric carcinomas in individuals born in Denmark. For every Greenlandic case, the Danish gastric carcinomas of equivalent gender, age (10-year interval) and year of diagnosis (5-year interval) were identified using the Danish Civil Registry System and the Danish Cancer Register. All these potential controls were listed on the basis of a random number, and the first number was selected as the control. To reduce logistic effort, Danish controls >40 years of age were recruited from Aarhus County (∼650 000 inhabitants), whereas controls below 40 years were collected from all over Denmark (∼5.2 million inhabitants). Paraffin-embedded tumour material was retrieved from pathology archives in 106 of 117 (90.6%) Greenlandic cases and in 106 of 117 Danish controls, thus collecting a total of 212 samples for analysis. Of the remaining 22 samples, 12 could not be traced (5 cases and 7 controls), 4 were unsuitable for EBV analysis due to lack of tissue (3 cases and 1 control), 4 were excluded due to a non-gastric carcinoma diagnosis (2 cases and 2 controls) and in 2 cases only tumour material from metastases was available (1 case and 1 control).

All cases were reclassified by expert histopathologists according to the WHO 2000 guideline for the diagnosis of gastric carcinomas (International Agency for Research on Cancer (IARC), 2000), sub-classified according to the Laurén classification and re-staged (pTNM). The pathologist was blinded to EBV status and country of origin. From the results of the original pathology report, the macroscopic location of the tumours was determined as proximal (proximal 2/3 of the stomach), distal (distal 1/3) or diffuse. Gastric carcinoma cells in paraffin sections were analysed for the presence of EBV-latent membrane protein-1 and EBERs using immunohistochemistry with antibody cocktail CS 1–4 (Dako, Glostrup, Denmark) and RNA *in situ* hybridisation using the INFORM EBER probe (Ventana Medical Systems, Illkirch, France), respectively, as described (Zhou *et al*, 2001) with modifications. EBV-positive tonsils with infectious mononucleosis and lymph nodes with Hodgkin's lymphoma were used as positive controls. These methods represent the ‘gold standard’ for EBV analysis in tissue sections, and can be expected to detect the majority of virally infected tumours, even in archive paraffin blocks.

The associations between tumour characteristics, and ethnicity and EBV status were evaluated by *P*-values obtained from logistic regression. Adjustments were made for sex, age (10-year interval) and year at diagnosis (5-year interval). Trend tests for variables with ordered categories were conducted by assigning the values 1, 2 and 3 to the ordered categories and by treating the variables as numerical in the regression analyses. A variation in the association between EBV-associated gastric carcinomas and ethnicity according to birth year was evaluated by tests for interaction. Significance level was set at 0.05.

The study was approved by the Danish data protection board and by the appropriate scientific ethical committees in both countries.

## Results

We examined 212 gastric carcinomas, 106 from Greenland and 106 from Denmark, respectively, from 135 men (64%) and 77 women (36%). The average age at diagnosis was 55.1 (range: 26–78) years among the Inuit and 55.8 (range: 22–79) years among the matched Danes (Table 1). In both populations, the frequency of EBV-associated gastric carcinomas was 8.5% (9 of 106) (Table 2), representing an odds ratio (OR) of 1.0 (CI: 0.4–2.7). EBV-associated gastric carcinomas were more common among men (15 of 135=11.1%) compared with women (3 of 77=3.9%), (*P*=0.19, OR=2.44; CI: 0.64–9.37) (Table 3). The associations between tumour characteristics, and ethnicity and EBV status are summarised in Tables 2 and 3. The EBV status was not associated with tumour location, but a distal location was more frequent among the Inuit (*P*=0.005; OR=0.40; CI: 0.21–0.76). Although the degree of lymphocytic infiltration was most frequently ‘low’ (119 of 212), it was significantly higher in EBV-associated gastric carcinomas than in non-EBV-associated gastric carcinomas (*P*=0.03; OR 2.36; CI 1.07–5.22). Tumour histology (WHO/Laurén) was not related to EBV status or ethnicity. Although undifferentiated gastric carcinomas were identified in patients from both Greenland and Denmark, none were of the lymphoepithelial type. We observed no temporal changes in either the proportion of EBV-GC or in the tumour location over the period (data not shown). The risk of having EBV-associated gastric carcinomas was not related to ethnicity; but the Inuit with EBV-positive cases were born earlier than the Danes with such tumours (*P*<0.01) (data not shown).

## Discussion

We found an equal proportion of EBV-associated gastric carcinomas in the Inuit and Danes, suggesting that the factors responsible for the high incidence of EBV-positive NPC and salivary gland carcinoma in Greenland do not influence the risk of EBV-associated gastric carcinomas.

The risk of NPC and salivary gland carcinoma are strikingly higher among the Inuit in Greenland compared with the Danes in Denmark, with standardised incidence ratios of ∼35 and 6, respectively (Friborg *et al*, 2003). There is also a clear increased familial risk of NPC and salivary gland carcinoma among the Inuit, with an eight-fold increased risk in first-degree relatives, which is among the highest reported for any cancer (Goldgar *et al*, 1994; Friborg *et al*, 2005). These high risks imply the existence of strong genetic or environmental risk factors for EBV-associated NPC and salivary gland carcinoma in the Inuit population. However, the similar proportion of EBV-associated gastric carcinomas in Greenland and Denmark argues against a general susceptibility to EBV-associated carcinomas among the Inuit. EBV-associated carcinomas of the nasopharynx and salivary glands in the Inuit are mainly of the lymphoepithelial type (Nielsen, 1986). It must be noted that, we did not identify an increased incidence of gastric carcinomas of this histopathological type in Greenland.

The proportion of EBV-associated gastric carcinomas in Greenland and Denmark (8.5%) is comparable with frequencies found worldwide (IARC working group, 1997; Burgess *et al*, 2002; Hjalgrim *et al*, 2008). Studies of EBV positivity in gastric carcinomas have produced varied results, with prevalence ranging from 2 to 15%. Some of this variation may be attributed to differences in sample collection and EBV detection. The advantages of our study include a population-based case collection, and optimal, uniform methods for EBV detection.

We did not find an increased proportion of EBV-associated gastric carcinomas in Greenland, a high-incidence region for other EBV-associated carcinomas, arguing against a general susceptibility to EBV-associated carcinomas among the Inuit.

## Figures and Tables

**Table 1:** The study population

	**Inuit *N*=106**	**Danes *N*=106**
Age at diagnosis (range)	55.1 (26–78)	55.8 (22–79)
Males/females	68/38	67/39
EBV positive (%)	8.5	8.5

EBV=Epstein–Barr virus.

**Table 2:** Tumour characteristics of gastric carcinomas according to ethnicity

	**Inuit *n*=106**		**Danes *n*=106**		
	***N***	**%**	***N***	**%**	***P*-value**
*EBV status*					0.99
EBV-positive gastric carcinoma	9	8.5	9	8.5	
EBV-negative gastric carcinoma	97	91.5	97	91.5	
					
*Material*					0.48
Biopsy	42	39.6	38	35.8	
Gastrectomy specimens	64	60.4	68	64.2	
					
*Location*					0.005^a^
Proximal	25	23.6	40	37.7	
Distal	55	51.9	37	34.9	
Diffuse	5	4.7	11	10.4	
Unknown	21	19.8	18	17.0	
					
*Histology (WHO)*					0.24^b^
Papillary	3	2.8	3	2.8	
Tubular	71	67.0	63	59.4	
Mucinous	4	3.8	1	0.9	
Signet-ring cell	20	18.9	25	23.6	
Undifferentiated	7	6.6	13	12.3	
Adenosquamous	1	0.9	1	0.9	
					
*Histology (Laurén)*					0.27^c^
Intestinal	72	67.9	67	63.2	
Diffuse	31	29.2	39	36.8	
Mixed	3	2.8	0	0	
					
*Differentiation of tubular type carcinomas*					0.25^d^
Good	11	15.5	6	9.5	
Moderate	37	52.1	31	49.2	
Poor	23	32.4	26	41.2	
					
*Degree of lymphocyte infiltration*					0.07^d^
Low	54	50.9	65	61.3	
Moderate	46	43.4	38	35.8	
Abundant	6	5.7	3	2.8	
					
*Pattern of lymphocyte infiltration*					0.99^e^
Lymphoepithelioma-like	0	0	0	0	
Nodular	22	20.7	22	20.7	
Diffuse	80	75.5	84	79.2	
Follicular infiltration	4	3.8	0	0	

EBV=Epstein–Barr virus; WHO=World Health Organisation.

Tubular adenocarcinomas were categorised as well differentiated (well-formed glands), moderately differentiated (intermediate between well differentiated and poorly differentiated) and poorly differentiated (highly irregular glands that are recognised with difficulty or single cells that remain isolated or are arranged in small clusters) according to WHO 2000. The pattern and degree of lymphocytic infiltration was assessed semi-quantitatively as either absent, mild, moderate or abundant infiltration by examining 10 medium power fields ( × 200 magnification). The pattern of lymphocyte infiltration was determined as lymphoepithelioma-like, nodular, diffuse or follicular.

a Non-distal *vs* distal location, samples with ‘unknown’ location not included.

b Tubular histology *vs* other types.

c Samples with ‘mixed’ histology not included.

d *P*-value represents trend test.

e Nodular infiltration *vs* other types.

**Table 3:** Tumour characteristics of gastric carcinoma according to EBV status

	**EBV-positive gastric carcinoma *n*=18**		**EBV-negative gastric carcinoma *n*=194**		
	***N***	**%**	***N***	**%**	***P*-value**
*Gender*					0.19
Males	15	83	120	62	
Females	3	17	74	38	
					
*Material*					0.98
Biopsy	8	44.4	72	37.1	
Gastrectomy specimens	10	55.6	122	62.9	
					
*Location*					0.33^a^
Proximal	6	33.3	59	30.4	
Distal	6	33.3	86	44.3	
Diffuse	2	11.1	14	7.2	
Unknown	4	22.2	35	18.0	
					
*Histology (WHO)*					0.12^b^
Papillary	0	0	6	3.1	
Tubular	15	83.3	119	61.3	
Mucinous	0	0	5	2.6	
Signet-ring cell	1	5.6	44	22.7	
Undifferentiated	2	11.1	18	9.3	
Adenosquamous	0	0	2	1.0	
					
*Histology (Laurén)*					0.23^c^
Intestinal	14	77.8	125	64.4	
Diffuse	3	16.7	67	34.5	
Mixed	1	5.6	2	1.0	
					
*Differentiation of tubular type carcinomas*					0.24^d^
Good	0	0	17	14.3	
Moderate	9	60.0	59	49.6	
Poor	6	40.0	43	36.1	
					
*Degree of lymphocyte infiltration*					0.03^d^
Low	6	33.3	113	58.2	
Moderate	9	50.0	75	38.7	
Abundant	3	16.7	6	3.1	
					
*Pattern of lymphocyte infiltration*					0.60^e^
Lymphoepithelioma-like	0	0	0	0	
Nodular	3	16.7	41	21.1	
Diffuse	15	83.3	149	76.8	
Follicular infiltration	0	0	4	2.1	

EBV=Epstein–Barr virus; WHO=World Health Organisation;

Tubular adenocarcinomas were categorised as well differentiated (well-formed glands), moderately differentiated (intermediate between well differentiated and poorly differentiated) and poorly differentiated (highly irregular glands that are recognised with difficulty or single cells that remain isolated or are arranged in small clusters) according to WHO 2000. The pattern and degree of lymphocytic infiltration was assessed semi-quantitatively as absent, mild, moderate or abundant infiltration by examining 10 medium power fields ( × 200 magnification). The pattern of lymphocyte infiltration was determined as lymphoepithelioma-like, nodular, diffuse or follicular.

a Non-distal *vs* distal location, samples with ‘unknown’ location not included.

b Tubular histology *vs* other types.

c Samples with ‘mixed’ histology not included.

d *P*-value represents trend test.

e Nodular infiltration *vs* other types.

## References

[bib1] Alberts SR, Kelly JJ, Lanier AP, Sacco F (2006) Occurrence of esophageal and gastric cancer in Alaska natives, 1969–2003. Alaska Med 48: 2–11 17042389

[bib2] Burgess DE, Woodman CB, Flavell KJ, Rowlands DC, Crocker J, Scott K, Biddulph JP, Young LS, Murray PG (2002) Low prevalence of Epstein-Barr virus in incident gastric adenocarcinomas from the United Kingdom. Br J Cancer 86: 702–704 11875729 10.1038/sj.bjc.6600107 PMC2375309

[bib3] Friborg J, Koch A, Wohlfarht J, Storm HH, Melbye M (2003) Cancer in Greenlandic Inuit 1973–1997: a cohort study. Int J Cancer 107: 1017–1022 14601064 10.1002/ijc.11502

[bib4] Friborg J, Wohlfahrt J, Koch A, Storm H, Olsen OR, Melbye M (2005) Cancer susceptibility in nasopharyngeal carcinoma families – a population-based cohort study. Cancer Res 65: 8567–8572 16166338 10.1158/0008-5472.CAN-04-4208

[bib5] Goldgar DE, Easton DF, Cannon-Albright LA, Skolnick MH (1994) Systematic population-based assessment of cancer risk in first-degree relatives of cancer probands. J Natl Cancer Inst 86: 1600–1608 7932824 10.1093/jnci/86.21.1600

[bib6] Hamilton-Dutoit SJ, Therkildsen MH, Neilsen NH, Jensen H, Hansen JP, Pallesen G (1991) Undifferentiated carcinoma of the salivary gland in Greenlandic Eskimos: demonstration of Epstein-Barr virus DNA by *in situ* nucleic acid hybridization. Hum Pathol 22: 811–815 1651284 10.1016/0046-8177(91)90210-g

[bib7] Hao Z, Koriyama C, Akiba S, Li J, Luo X, Itoh T, Eizuru Y, Zou J (2002) The Epstein-Barr virus-associated gastric carcinoma in Southern and Northern China. Oncol Rep 9: 1293–1298 12375037

[bib8] Hjalgrim H, Friborg J, Melbye M (2008) In Human Herpesviruses – Biology, Therapy, and Immunoprophylaxis, Arvin, Campadelli-Fiume, Mocarski, Moore, Roizman, Whitley, Yamanishi (eds) pp 947–948. Cambridge University Press: Cambridge 21348071

[bib9] IARC working group (1997) Epstein-Barr Virus and Kaposi's Sarcoma Herpesvirus/Human Herpesvirus 8. World Health Organization Internationl Agency for Research on Cancer: Lyon

[bib10] Imai S, Koizumi S, Sugiura M, Tokunaga M, Uemura Y, Yamamoto N, Tanaka S, Sato E, Osato T (1994) Gastric carcinoma: monoclonal epithelial malignant cells expressing Epstein-Barr virus latent infection protein. Proc Natl Acad Sci USA 91: 9131–9135 8090780 10.1073/pnas.91.19.9131 PMC44761

[bib11] International Agency for Research on Cancer (IARC) (2000) Pathology and genetics of tumours of the digestive system. In: International Agency for Research on Cancer (IARC) Stanley R. Hamilton and Lauri A. Aaltonen (eds) pp 35–52. World Health Organization Classification of Tumours: Lyon

[bib12] Nielsen NH (1986) Cancer incidence in Greenland. Arctic Med Res 43: 1–168 3300680

[bib13] Parkin DM, Bray F, Ferlay J, Pisani P (2005) Global cancer statistics, 2002. CA Cancer J Clin 55: 74–108 15761078 10.3322/canjclin.55.2.74

[bib14] Shibata D, Weiss LM (1992) Epstein-Barr virus-associated gastric adenocarcinoma. Am J Pathol 140: 769–774 1314023 PMC1886378

[bib15] Storm HH, Michelsen EV, Clemmensen IH, Pihl J (1997) The Danish Cancer Registry – history, content, quality and use. Dan Med Bull 44: 535–539 9408738

[bib16] Zhou XG, Sandvej K, Li PJ, Ji XL, Yan QH, Zhang XP, Da JP, Hamilton-Dutoit SJ (2001) Epstein-Barr virus (EBV) in Chinese pediatric Hodgkin disease: Hodgkin disease in young children is an EBV-related lymphoma. Cancer 92: 1621–1631 11745241 10.1002/1097-0142(20010915)92:6<1621::aid-cncr1488>3.0.co;2-p

